# New Insight into Taxonomy of European Mountain Pines, *Pinus mugo* Complex, Based on Complete Chloroplast Genomes Sequencing

**DOI:** 10.3390/plants10071331

**Published:** 2021-06-29

**Authors:** Joanna Sokołowska, Hanna Fuchs, Konrad Celiński

**Affiliations:** 1 Department of Genetics, Institute of Experimental Biology, Faculty of Biology, School of Natural Sciences, Adam Mickiewicz University, Poznań, Uniwersytetu Poznańskiego 6, 61-614 Poznań, Poland; joanna.sokolowska@amu.edu.pl; 2 Institute of Dendrology, Polish Academy of Sciences, Parkowa 5, 62-035 Kórnik, Poland; hkijak@man.poznan.pl

**Keywords:** Pinaceae, European mountain pines, closely related taxa, next-generation sequencing

## Abstract

The *Pinus mugo* complex is a large group of closely related mountain pines, which are an important component of the ecosystems of the most important mountain ranges, such as the Alps, Carpathians and Pyrenees. The phylogenetic relationships between taxa in this complex have been under discussion for many years. Despite the use of many different approaches, they still need to be clarified and supplemented with new data, especially those obtained with high-throughput methods. Therefore, in this study, the complete sequences of the chloroplast genomes of the three most recognized members of the *Pinus mugo* complex, i.e., *Pinus mugo*, *Pinus rotundata* and *Pinus uncinata*, were sequenced and analyzed to gain new insight into their phylogenetic relationships. Comparative analysis of their complete chloroplast genome sequences revealed several mutational hotspots potentially useful for the genetic identification of taxa from the *Pinus mugo* complex. Phylogenetic inference based on sixteen complete chloroplast genomes of different coniferous representatives showed that pines from the *Pinus mugo* complex form one distinct monophyletic group. The results obtained in this study provide new and valuable omics data for further research within the European mountain pine complex. They also indicate which regions may be useful in the search for diagnostic DNA markers for the members of *Pinus mugo* complex and set the baseline in the conservation of genetic resources of its endangered taxa.

## 1. Introduction

The *Pinus mugo* complex is a large and polymorphic complex of closely related pines native to the main mountains of Europe, including the Pyrenees, the Alps and the Carpathians [1,2]. Some researchers indicate that in this group there may be even more than a hundred endemic forms classified into various taxonomic ranks, i.e., species, subspecies or varieties [1]. However, among them only three taxa, i.e., *Pinus mugo* subsp. *mugo*, *Pinus mugo* subsp. *rotundata* and *Pinus mugo* subsp. *uncinata,* are more widely known and thoroughly studied. These taxa differ in some phenotypic features, geographical distribution or preferred habitat. *Pinus mugo* subsp. *mugo*, also known as *Pinus mugo* Turra (dwarf mountain pine) or *Pinus mugo sensu stricto*, is a shrub with long, curved branches, reaching up to 3.5 m in height. The taxa has a wide geographical range, including the Alps, Pyrenees, Carpathians and Balkans [3], but most often occur in the higher parts of the mountains at an altitude of 1600–2200 m.a.s.l. [4]. *Pinus mugo* subsp. *rotundata*, identified by some researchers as a synonym for *Pinus uliginosa* Neumann (peat-bog pine), is usually a tree-shaped form, with a geographical range limited to peat bog areas of Poland, the Czech Republic and Germany [5]. *Pinus mugo* subsp. *uncinata* known as *Pinus uncinata* Rammond (mountain pine) is a tree with a height of 12–20 m, occurring in the Pyrenees and the western Alps, as well as the Central Massif and the Iberian System [1,2]. 

These three taxa are considered to be independent species or subspecies inside *Pinus mugo* complex known also *Pinus mugo* Turra *sensu lato* [1,2,6]. The International Union for Conservation of Nature (IUCN) has defined the status of *Pinus mugo* subsp. *mugo* and *Pinus mugo* subsp. *uncinata* as least concern (LC), while *Pinus mugo* subsp. *rotundata* is identified as endangered (EN) [7]. However, conservation of these taxa can be difficult for a number of reasons. One of them is the problematic identification and classification of atypical individuals to specific taxa, especially in sympatric populations. In such populations, natural and uncontrolled gene flow is observed, as well as the formation of hybrid individuals with a phenotype intermediate between those of parent taxa [8,9,10,11]. Another serious problem is the functioning of synonyms in the scientific literature, which probably (but not for sure) refer to the same taxon, e.g., *Pinus mugo* subsp. *rotundata* also appears in the literature as *Pinus uliginosa* but can also be understood as *Pinus* × *rhaetica* (as a hybrid of *Pinus sylvestris* × *Pinus mugo*) [2]. The relations between these synonyms require urgent and detailed analyses, especially since *Pinus uliginosa* is the most endangered pine in Poland, as the number of individuals is gradually declining [12,13].

Until now, representatives of the *Pinus mugo* complex have been the subject of many different studies, including needle biometric analyses [14,15,16], characteristics of allozyme variability [17,18], patterns of genetic diversity distribution in the geographical aspect [19,20,21], gene flow and hybridization [22,23,24], molecular cytogenetics or flow cytometric analyses [25,26]. Some of the most important aspects so far undertaken by researchers were also attempts to establish relations between taxa in this complex [27,28] or searching for diagnostic features or additional determinants allowing for their unambiguous and simple differentiation [29,30,31]. However, based on the results obtained so far, it is extremely difficult to draw consistent and unambiguous conclusions, especially far-reaching ones. On the one hand, numerous studies indicate differences in gene expression products such as volatiles [32], essential oils [33] or seed protein patterns [31] between *P. mugo*, *P. uliginosa* and *P. uncinata*. On the other hand, other studies indicate that taxa from the *Pinus mugo* complex share common chloro- and mitotypes [20,34], have a complex genetic background and are generally characterized by a conservative organization of genomes [25,26].

Despite many studies on *P. mugo*, *P. rotundata* and *P. uncinata*, their origin, species distinctiveness and taxonomic status within the *Pinus mugo* complex as well as the identification of additional diagnostic determinants for them require further analysis.

The use of complete chloroplast genome sequences obtained by high throughput techniques could greatly help in this regard, by significantly increasing the phylogenetic resolution and providing new insight into the taxonomic relationships within this complex. This approach is particularly recommended in the case of closely related taxa, where the use of whole chloroplast genomes as one super-barcode should bring better resolution effects than the use of one or even several universal or specific DNA barcodes, which may be too little variable in a given group of plants [35]. This approach seems to be particularly relevant in the case of the *Pinus mugo* complex, where the analysis of several core, supplementary and candidate barcode regions failed to distinguish these taxa at the DNA level [30]. A detailed comparative analysis of the complete sequences of chloroplast genomes was successfully used in the research, among others, in *Pseudolarix* and *Tsuga* [36], *Corylus* [37], *Magnolia* [38] or *Quercus* [39] as well as many others plant taxa.

Therefore, the main objectives of our research were: (1) sequencing, analysis and characterization of the entire genomes of *P. mugo*, *P. rotundata* and *P. uncinata* chloroplasts; (2) comparative analysis of the obtained complete chloroplast genome sequences with previously published data for other members of the *Pinus* genus, especially those for *P. sylvestris*; (3) identifying and selecting mutation regions (hot spots) in chloroplast genomes potentially useful in identifying *Pinus mugo* taxa; and (4) performing a phylogenetic inference about the relatedness of three closely related taxa of the *Pinus mugo* complex based on the complete sequences of the chloroplast genomes as well as selected regions.

Our results gain new insight into the taxonomy of this highly polymorphic group of closely related taxa, significantly increasing phylogenetic resolution and providing new genomic resources for further taxonomic research and as a baseline to take conservation measures for this ecologically important group of European mountain pines. 

## 2. Results and Discussion

### 2.1. General Features of P. mugo, P. rotundata and P. uncinata Chloroplast Genomes

Chloroplast genomes are typically about 150 kb in length and have a fairly distinctive quadripartite structure consisting of a large single copy (LSC), a small single copy (SSC) regions and two inverted repeats (IR) that separate them. Usually these repeats (IRa and IRb) are about 20-30 kb long, although in the case of the Pinaceae they are extremely reduced-to fragments sometimes even within 400 bp. The number of genes annotated in chloroplast genomes is variable, ranging from 63 to even 209 genes, although usually it does not exceed the range of 110 and 130 [39,40,41].

**Figure 1:**
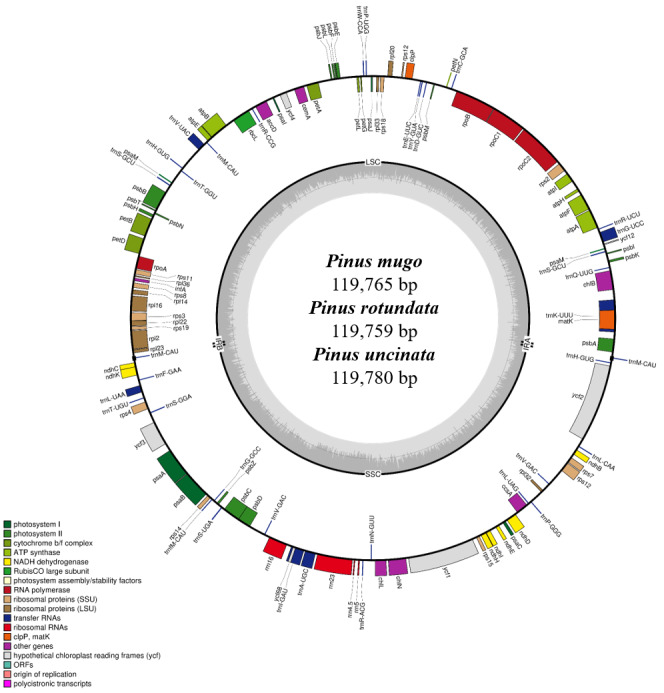
Gene maps of the three *Pinus* chloroplast genomes. The genes inside the circle are transcribed clockwise, and those outside are transcribed counterclockwise. Genes of different functions are color coded. The darker gray in the inner circle shows the GC content, while the lighter gray shows the AT content. IRA, IRB, inverted repeats; LSC, large single copy region; SSC, small single copy region.

The length of complete chloroplast genomes of three closely related *P. mugo*, *P. rotundata* and *P. uncinata* analyzed in this study is comparable and amounts to 119,765 bp, 119,759 bp and 119,780 bp, respectively for these taxa (Figure 1 and Table 1). Chloroplast genomes of representatives of the *Pinus mugo* complex are circular molecules with a typical quadripartite structure consisting of a large single copy (LSC), a small single copy (SSC) and two very short inverted repeated IRs (IRa and IRb). The length of the LSC region ranges from 65,879 bp for *P. rotundata* to 65,899 bp for *P. uncinata* and *P. mugo* while the length of the SSC region ranges from 53,164 bp for *P. mugo*, 53,168 bp for *P. rotundata* to 53,169 bp for *P. uncinata*. The IR regions, on the other hand, are strongly reduced and, in the case of the representatives of the *Pinus mugo* complex, they are only 365 bp, which is one of the shortest so far described in the Pinaceae family. For comparison, the IR length in *Pinus taeda* (KC427273) is 485 bp, and for *Pinus sylvestris* (KR476379), *Pinus densiflora* (MK285358) or *Pinus yunnanensis* (MK007968) is exactly 495 bp [42,43]. For other species, differences in IR lengths are also observed, and several studies report that contraction and expansion of IR regions are quite common phenomena in plants [44]. Moreover, it happens that in some species these regions are completely lost [45,46,47]. It is postulated that the contraction and expansion of the IR regions play a major role in evolution and are responsible for altering the length of genomic sequences.

The plastomes of *P. mugo*, *P. rotundata* and *P. uncinata* contain 121 genes, including 115 unique genes (excluding duplicate ones), 73 protein-coding genes, 36 transfer RNA genes, and four ribosomal RNA genes (Figure 1, Table 1). Five genes are duplicated, i.e., *psaM* (x2), *trnH-GUG* (x2), *trnM-CAU* (x3), *trnS-GCU* (x2) and *trnV-GAC* (x2). The functional classification of these genes is presented in Supplementary Table S1. The total content of GC is 38.5% and there are no differences in this parameter between the analyzed taxa. Likewise, there are no significant differences in the length of the protein coding sequences (60,339 bp), the total and unique number of genes (121 and 115, respectively) or the number of rRNA and tRNA genes (4 and 36, respectively). Due to the uniform gene number, order and their names, annotated chloroplast genomes of these three taxa from the *Pinus mugo* complex are presented on one circular map (Figure 1). 

Our results obtained in this study are fully consistent with those previously published for other *Pinus* representatives, i.e., *P. sylvestris* (KR476379) or *P. densiflora* (MK285358) [42] in terms of genome size, total coding length, and protein coding length, as well as number of predicted genes or GC content (Table 1). There are only slight differences in genomic features between *Pinus* taxa and *Larix* or *Abies* taxa. They mainly concern the size of the genome and the number of genes. Taxa of the genera *Larix* and *Abies* have slightly longer genomes and fewer genes than representatives of the genus *Pinus*.

### 2.2. Genome Comparative Analysis and Identification of Divergent Hotspots

**Figure 2:**
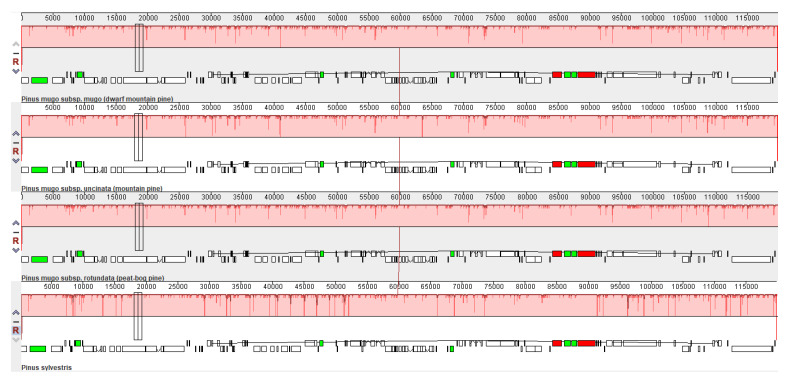
MAUVE alignment of three *Pinus mugo* complex representatives; *P. mugo* subsp. *mugo*, *P. mugo* subsp. *uncinata*, *P. mugo* subsp. *rotundata*. The *Pinus sylvestris* chloroplast genome is shown at bottom as a reference. Within each of the alignments, local collinear blocks are represented by blocks of the same color connected by lines.

The complete sequences of the *P. mugo*, *P. rotundata* and *P. uncinata* chloroplast genomes were aligned with the complete *P. sylvestris* chloroplast genome (KR476379) to compare the organization of their genomes (Figure 2). *Pinus sylvestris* was chosen as the reference taxon closest to this complex but not belonging to it. Figure 2 shows only one locally collinear block (LCB) between all analyzed chloroplast genomes, which suggests a high level of similarity in genome organization between the analyzed *Pinus* taxa. 

In summary, whole-genome alignment of the chloroplast sequences did not reveal any rearrangement or inversion events among *Pinus* chloroplast genomes, and confirmed the close evolutionary relationships between all analyzed taxa (both those belonging to the *Pinus mugo* complex and not). Our results are fully consistent with earlier studies on *Pinus* species [42], in which the gene content and order of the *P. densiflora* chloroplast genome were similar to four other pines, i.e., *P. sylvestris*, *P. thunbergii*, *P. tabuliformis* and *P. taeda* [42]. 

The K2p distance values calculated as an estimator of evolutionary divergence (Table 2) differ between *Pinus* taxa from 0.000259 in a pair of *P. mugo* and *P. uncinata* to 0.00318 in a pair of *P. uncinata* and *P. sylvestris*, with an average of 0.001741 for all four analyzed *Pinus* taxa.

**Figure 3:**
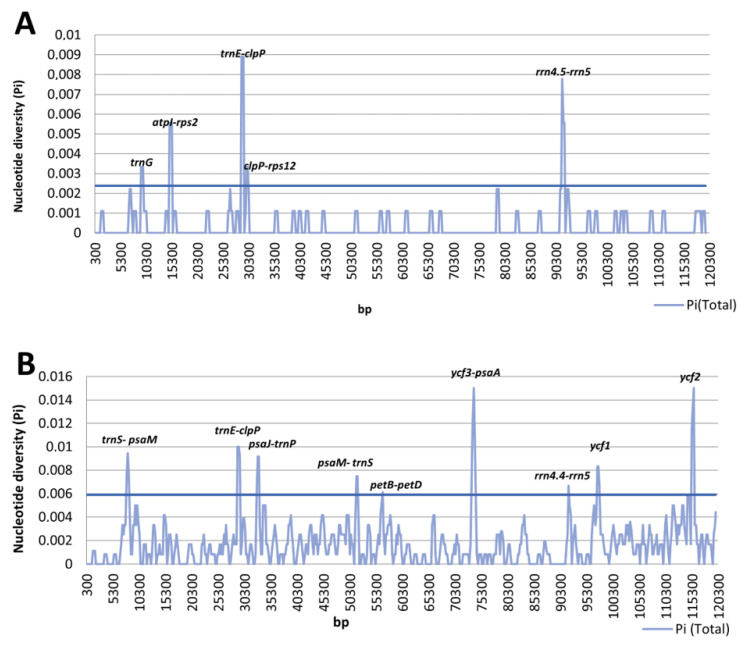
Sliding window analysis of the whole chloroplast genomes. Window length: 600 bp; step size: 200 bp. X-axis: position of the midpoint of a window. Y-axis: nucleotide diversity of each window. (**A**) Pi between three the *Pinus mugo* complex representatives. (**B**) Pi among the *Pinus mugo* complex representatives and *Pinus sylvestris*. The horizontal line on the graph sets the threshold separately for the *Pinus mugo* complex representatives (0.00238) and separately for the *Pinus mugo* complex representatives and *Pinus sylvestris* (0.00589).

DnaSP was used to perform two sliding window analyses in order to identify mutational regions. One analysis concerned only three taxa from the *Pinus mugo* complex (Figure 3A), while the other, apart from *P. mugo*, *P. rotundata* and *P. uncinata*, also included *P. sylvestris* (Figure 3B). 

The results in Figure 3A clearly show that for the *Pinus mugo* complex taxa there were five divergent hotspots with a high Pi value (>0.00238), i.e., *trnG*, *atpI-rps2*, *trnE-clpP*, *clpP-rps12*, and *rrn4.5-rrn5*. For the second combination, taxa from the *Pinus mugo* complex and *P. sylvestris*, a total of nine unique mutational regions with a high Pi value (>0.00589) were detected, i.e., *trnS-psaM*, *trnE-clpP*, *psaJ-trnP*, *psaM-trnS*, *petB-petD*, *ycf3-psaA*, *rrn4.5-rrn5*, *ycf1* and *ycf2* (Figure 3B). The average value of nucleotide diversity (Pi) was 0.00036 and 0.00174 for the *Pinus mugo* complex taxa and for the *Pinus mugo* taxa together with *P. sylvestris*, respectively. This result is in line with expectations because the second combination included more distant pines, not just three closely related taxa. A similar relationship was found also in the case of other species [49].

**Figure 4:**
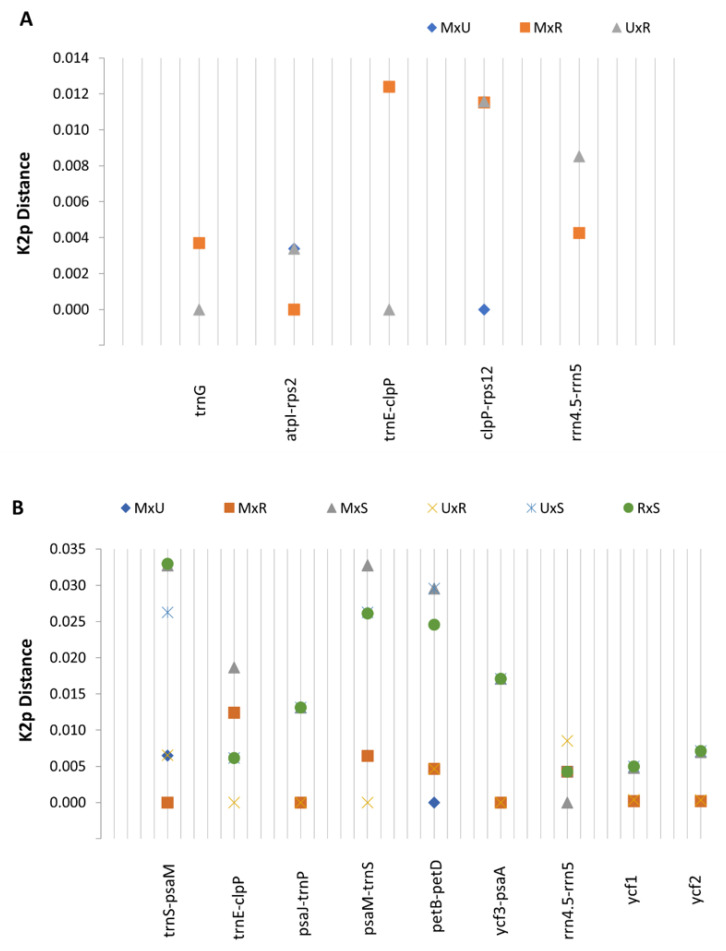
K2p distance for selected hotspot regions separately for the *Pinus mugo* complex representatives (**A**) and taxa for the *Pinus mugo* complex and *P. sylvestris* (**B**). Abbreviations: M, *Pinus mugo*; R, *Pinus rotundata*; U, *Pinus uncinata*; S, *Pinus sylvestris*.

Pairwise distance analysis for the highly variable regions (Figure 4A,B) showed that the highest K2p distance between taxa from the *Pinus mugo* complex is between *P. mugo* and *P. rotundata* (0.01239) in the *trnE-clpP* region (Figure 4A). In turn, the highest K2p distance between *P. sylvestris* and any taxon from the *Pinus mugo* complex (Figure 4B) is 0.03298 and concerns the *trnS-psaM* region and the *P. sylvestris* vs. *P. rotundata*. Overall, a detailed pairwise distance analysis revealed what values of discrepancy and in which regions of the chloroplast genome sequence can be expected between the analyzed taxa pairs.

Chloroplast DNA regions selected in this study can be preferentially used as specific barcodes for further studies of *Pinus mugo* taxonomy. A species-specific barcode is defined as a fragment of a DNA sequence with a sufficiently high mutation rate to enable the species to be identified within a given taxonomic group [35]. The *ycf1* and *ycf2* regions seem of particular interest in this regard for the genus *Pinus*. Several studies show that the *ycf1* region in particular has extremely high discriminatory power in some genera and much greater potential than the commonly used universal core barcodes [30,50,51].

### 2.3. Simple Sequence Repeats Analysis 

Simple sequence repeats (SSRs or microsatellites) are very often used in population, ecological and conservation genetics as effective molecular markers. Their most important advantages are the high level of genetic polymorphism detected by them and wide distribution throughout the genome of chloroplasts, as well as trouble-free amplification, fast electrophoretic separation or objective and simple statistical analysis [52,53,54,55].

In this study, a total of fifty-nine SSRs with a length of at least 10 bp were detected in the chloroplast genomes of three members of the *Pinus mugo* complex. The number of detected SSR loci ranged slightly from nineteen in *P. uncinata* to twenty in *P. mugo* and *P. rotundata* and was similar to *P. sylvestris* (22 microsatellites) but much lower than that found recently with another pine, *Pinus taeda* (151) [56]. 

Interestingly, the identified differences in the number of SSRs between the four analyzed taxa hypothetically allow these taxa to be distinguished using microsatellite loci. A detailed analysis of the number and distribution of SSRs brings very interesting results. For *P. mugo* and *P. uncinata*, we found a microsatellite between 54,429 and 54,438 bp and between 54,428 and 54,437 bp, respectively, which was not observed in the genomes of *P. rotundata* or *P. sylvestris*. Similarly, in the case of *P. mugo* and *P. rotundata*, we found the presence of a microsatellite repeat between 44,949 and 44,961 bp and 44,940 and 44,954 bp, respectively, which is not present in the chloroplast genome of *P. uncinata*. A comparison of the 100,883-100,892 bp region in *P. rotundata* with the 100,844-100,853 region in *P. sylvestris* reveals that these taxa differ in the repeat motif; *P. sylvestris* has an A repeat, and *P. rotundata* has a T repeat. Moreover, to a similar extent, no microsatellite repetitions were found in the other two taxa, i.e., *P. mugo* and *P. uncinata*. Most of the SSRs identified in this study (47/59) were located in the intergenic distance region (IGS) (Table 3). The most common microsatellite repeat motif was mononucleotide (84.75%), followed by dinucleotide (10.17%) and compound (5.08%). Our results are fully consistent with the observations from other previously conducted studies in which SSRs in chloroplast genomes have a motif composed mainly of short polyadenine (polyA) or polythymine (polyT) repeats and much less often contain guanidine (G) or cytosine (C) tandem repeats [38,56].

The SSRs identified in this study can be used for further research on the representatives of the *Pinus mugo* complex, i.e., *P. mugo*, *P. rotundata* and *P. uncinata*, and to characterize their genetic resources. The SSRs described in this study can potentially be used to distinguish taxa in the *Pinus mugo* complex and also complement other microsatellite loci used so far for this purpose [57,58].

### 2.4. Phylogenetic Inference 

The phylogenesis of many different groups of plants was determined by analyzing the sequences of both the complete genome of chloroplasts and selected regions [59,60,61,62]. In this study, we were particularly interested in the relationships within the *Pinus mugo* complex between three closely related taxa, as the phylogeny of the genus *Pinus* is well known. Therefore, phylogenetic trees were constructed using the ML and Bayes algorithms using the nucleotide sequences of the chloroplast genomes of sixteen taxa representing the two main conifer families, Pinaceae and Podocarpaceae (Table 4). We used two datasets. The first involved alignment of entire chloroplast genome sequences, while the second was based on alignment of the highly variable *ycf1* gene only. In many previous studies, researchers indicate its very high level of genetic diversity, useful in phylogenic analyses [30,51,63]. 

**Figure 5:**
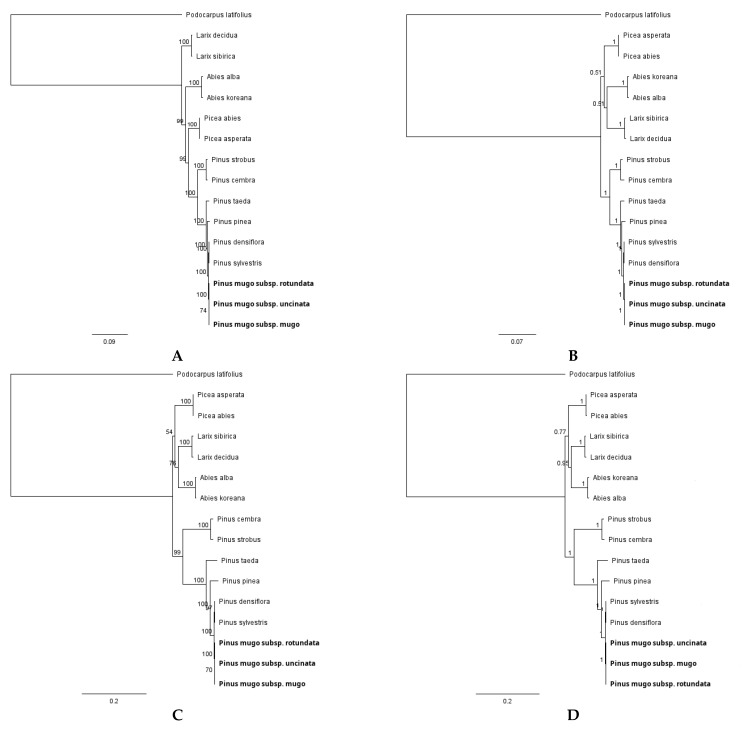
Phylogenetic relationships between sixteen conifers taxa based on complete sequences of chloroplast genomes (**A**,**B**) inferred from ML and BI analyses, respectively, and only on *ycf1* (**C**,**D**) also inferred from ML and BI analyses, respectively.

As shown in Figure 5, both obtained ML and Bayesian phylogenetic trees clearly indicated that *P. mugo*, *P. rotundata* and *P. uncinata* belonging to the *Pinus mugo* complex formed a separate cluster within the *Pinus* genus. Although phylogenetic reconstruction was not the main focus of this work, the overall topology of the trees obtained here (regardless of the data set and analysis methods used) was not surprising, and is consistent with the well-known and widely accepted division of the Pinaceae family into basic genera, i.e., *Picea*, *Larix*, *Abies* and *Pinus*. Additionally, in the genus *Pinus*, the analyzed pine taxa formed two separate clades. One clade consisted of *Pinus strobus* and *Pinus cembra* belonging to the subgenus *Strobus*, while the other clade consisted of taxa included in the subgenus *Pinus*, i.e., *Pinus taeda*, *Pinus pinea*, *Pinus densiflora*, *P. sylvestris* as well as three closely related taxa from of the *Pinus mugo* complex; *P. mugo*, *P. rotundata* and *P. uncinata*. It is worth noting that in the ML and BI trees, most of the nodes had 100% bootstrap support and 1.0 Bayesian posterior probability (Figure 5). *Podocarpus latifolius* from the Podocarpaceae family, as predicted, was outside the main group of taxa from the Pinaceae family.

## 3. Materials and Methods

### 3.1. Sampling, DNA Extraction and Genomic Library Preparation 

Fresh and healthy needles of the three most recognized members of the *Pinus mugo* complex were collected as follows: *Pinus mugo* subsp. *uncinata* (hereinafter referred to for short as *Pinus uncinata)* (collection number 1347) from the Dendrological Garden of University of Life Sciences, Poznań, Poland (52°25′37′′ N, 16°53′48′′ E); *Pinus mugo* subsp. *rotundata* (hereinafter referred to for short as *Pinus rotundata*) from the Great Peat Bog of Batorów located in Stołowe Mountains National Park, Poland (50°15′ 42.48′′ N, 16°8′31.92′′ E) and finally *Pinus mugo* subsp. *mugo* (hereinafter referred to for short as *Pinus mugo*) from the Tatra National Park (UNESCO Biosphere Reserve), Poland (49°10′0″ N, 19°55′0″ E). The collected needles were stored at 4 °C, until DNA extraction. Genomic DNA was isolated using the CTAB method [64]. The quality and integrity of isolated DNA were determined using agarose gel electrophoresis and measurement on a NanoDrop spectrophotometer (Thermo Fisher Scientific, Carlsbad, CA, USA). The genomic library was prepared according to the manufacturer’s recommendations with protocol: Ion Xpress™ Plus gDNA Fragment Library Preparation, using Ion Xpress Plus Fragment Library Kit (Pub. No. MAN0009847) (ThermoFisher Scientific, Waltham, MA, USA). The 100 ng of total genomic DNA was fragmented using Ion Shear Plus Reagents with 8 min incubation time at 37 °C, targeting fragments length of 200–300 bp. Then, the fragmented DNA was purified using 1.8× sample volume of Agencourt™ AMPure™ XP Reagent. The fragment size was checked by 2200 Tapestation Bioanalyzer and Agilent™ High Sensitivity DNA Kit (Agilent Technologies, Waldbronn, Germany), according to protocol: Agilent HS D1000 ScreenTape System Quick Guide. For *Pinus uncinata*, the adapters ligation was conducted for reaction setup for non-barcoded libraries using Ion Plus Fragment Library Kit Adapters. For *P. mugo* and *P. rotundata,* the adapters ligation was conducted for reaction setup for barcoded libraries using the Ion Xpress™ Barcode Adapters Kit. AMPure purification was performed after ligation using a 1.2× sample volume of Agencourt™ AMPure™ XP Reagent (ThermoFisher Scientific, Waltham, MA, USA) for 200–300-base-read library size. The size selection procedure was performed on the E-Gel™ SizeSelect™ 2% Agarose Gel, then the libraries were amplified and purified using a 1.2x sample volume of Agencourt™ AMPure™ XP Reagent (ThermoFisher Scientific, Waltham, MA). Quality and length analysis was conducted using 2200 Tapestation Bioanalyzer (Agilent Technologies Waldbronn, Germany). Chloroplast genomes are typically about 150 kb in length and have a fairly distinctive quadripartite

### 3.2. Next Generation Sequencing 

The genomic library was diluted to 100 pM. The concentration was measured on the Qubit™ 2.0 Fluorometer using Qubit™ dsDNA HS Assay Kit (Pub. No. MAN0002326 Revision: B.0) (Life Technologies). The *P. uncinata* template preparation was performed according to protocol: Ion PGM™ Hi-Q™ View OT2 Kit (Cat. No. A29900, Pub. No. MAN0014580 Rev. C.0). *P. mugo* and *P. rotundata* templates preparation were performed according to protocol: Ion 540™ Kit – OT2 (Cat. No A27753 Pub. No. MAN0010852 Rev. E.0). Evaluation of the templated Ion Sphere™ Particles (ISPs) was conducted using Ion Sphere™ Quality Control Kit (Cat.No. 4468656), according to protocol Ion Sphere™ Assay on the Qubit ™ 2.0 Fluorometer (Pub. No. MAN0016387 Revision A.0) (ThermoFisher Scientific, Waltham, MA, USA). *P. uncinata* genome sequencing was conducted on Ion 318™ Chip v2 BC by Ion Personal Genome Machine™ (PGM™) System (Thermo Fisher Scientific, Waltham, MA, USA) according to manufacturer’s recommendations using protocol: Ion PGM™ Hi-Q™ View Sequencing Kit user guide (Cat. No. A30044, Pub. No. MAN0014583). Then, *P. mugo* and *P. rotundata* genome sequencing was conducted on Ion 540™ Chip by GeneStudio™ S5 System (Thermo Fisher Scientific, Waltham, USA) according to manufacturer’s recommendations using protocol: Ion 540™ Kit – OT2 User Guide (Cat. No A27753, Pub. No MAN0010850, Rev. D). 

### 3.3. Chloroplast Genomes Assembly and Gene Annotation 

BBDuk Adapter/Quality Trimming V. 35.82 available in Geneious Prime 2020.2.5 [65] was used to filter low quality reads and trim low quality ends and adapters. The filtered reads were de novo assembled into contigs using Geneious Assembler on default options with merging homopolymer variants. Contigs were mapped to the reference genome *Pinus sylvestris* (NC_035069.1) using Geneious Mapper with minimum mapping quality: 30. Reads, which mapped to the reference genome, were used to assemble de novo the complete chloroplast genome sequences of *P. mugo*, *P. rotundata* and *P. uncinata*. Assembled genomes were initially annotated using CPGAVAS2, an integrated plastome sequence annotator [66], and GeSeq [67] and compared to the *Pinus sylvestris* (RefSeq: NC_035069.1) reference sequence. Location of large single copy region (LSC) and small single copy region (SSC) as well as calculation of GC content was carried out in Geneious Prime 2020.2.5 [65] by comparison with homologous sequences available to other *Pinus* representatives. Transfer RNAs were also checked with tRNAscan-RE v2.0.3. [68] incorporated in GeSeq [67] using default settings. OrganellarGenomeDRAW (OGDRAW) version 1.3.1 [69] was used to draw a circular map chloroplast genome of *P. mugo*, *P. rotundata* and *P. uncinata*. The complete sequences of the chloroplast genomes of these three taxa mentioned above have been deposited in GenBank under the following accession numbers: MZ333466 for *Pinus mugo* subsp. *mugo*; MZ333465 for *Pinus mugo* subsp. *rotundata* and MZ333464 for *Pinus mugo* subsp. *uncinata*. 

### 3.4. Genome Comparative Analysis and Identification of Divergent Hotspots 

In order to study genome-wide evolutionary dynamics among *P. mugo*, *P. rotundata* and *P. uncinata* from the *Pinus mugo* complex and to search evolutionary events such as gene loss, duplication, rearrangements and translocations, multiple alignments were made using progressive MAUVE algorithm with default settings via MAUVE [70] plugin v1.1.1 available in Geneious Prime 2020.2.5 [65]. The complete sequences of the *P. mugo*, *P. rotundata* and *P. uncinata* chloroplast genomes were compared with this previously published sequence for *Pinus sylvestris* (KR476379), which is the nearest taxa to the *Pinus mugo* complex, but does not belong to it (Table 1). Evolutionary divergence between the three representatives of the *Pinus mugo* complex and *P. sylvestris* was estimated by calculating genetic distances using the Kimura 2-parameters (K2p) evolution model [46,71] implemented in MEGA X [48].

Identification of divergent hotspots was performed separately only for the representatives of the *Pinus mugo* complex and for those representatives and *P. sylvestris* on the basis of three and four complete sequences of chloroplast genomes, respectively. The relevant chloroplast genomes were aligned using MAFFT v7.450 with default options [72], and then nucleotide diversity (Pi) was calculated through sliding window analysis using DnaSP version 6 [73]. The window length was set to 600 bp, with a step size 200 bp. The diversity thresholds for the *Pinus mugo* complex (0.00238) and for the *Pinus mugo* complex and together with *P. sylvestris* (0.00589) were calculated by sum of the average and double the standard deviation [74]. Regions with levels of nucleotide diversity higher than these thresholds were recommended as highly variable regions. Pairwise distance was also determined for these regions using the Kimura 2-parameters (K2p) evolution model [46,71] implemented in MEGA X [48].

### 3.5. Identification of Simple Sequence Repeats 

Simple sequence repeats (SSRs) in chloroplast genomes of *Pinus mugo* complex representatives and *Pinus sylvestris* were detected by MIcroSAtellite (MISA) [75], with the following parameters set at ≥10 for mononucleotides, 6≥ for dinucleotides and ≥5 for tri-, tetra-, penta- and hexanucleotides, respectively.

### 3.6. Phylogenetic Inference 

Phylogenetic inferences were constructed by maximum likelihood (ML) and Bayesian inference (BI) were constructed by maximum likelihood (ML) analysis using sixteen complete sequences of chloroplast genomes of various conifers representatives (including data obtained in this study for *P. mugo*, *P. rotundata* and *P. uncinata*). The list of taxa included in the study, along with GenBank accession numbers, is given in Table 4. In order to better explain the topology of the tree, both closely related taxa from the Pinaceae family, such as *Pinus*, and more distant taxa from the genus *Abies*, *Larix* and *Picea*, were selected. The outgroup was *Podocarpus latifolius* from the Podocarpaceae family.

Complete chloroplast genomes were aligned with MAFFT v7.450 using default settings [51]. A General Time Reversible + Gamma nucleotide substitution model (GTR + G) was selected according to Akaike’s information criterion (AIC) [76] with MEGA X [48], as the best substitution model for the ML and BI analyses. The ML analyses were conducted in RaxML v8.2.11 [77], with 1000 rapid bootstrap replicates along with a search for the best-scoring ML tree in every run and parsimony random seed set to 10.

BI analyses were conducted using MrBayes v 3.2.6 [78,79]. The Markov Chain Monte Carlo (MCMC) algorithm was run for 100,000 generations and the trees were sampled every 100 generations. The first 25% of the trees were discarded as a burn-in, and remaining trees were used to generate the consensus tree, including clade posterior probability (PP). Convergence was determined by examining the average standard deviation of the split frequencies (<0.01).

## 4. Conclusions

In this study, we aimed to increase the phylogenetic resolution within the European mountain pine complex using, for the first time, a detailed comprehensive comparative analysis of the complete chloroplast genome sequences of the three main representatives of this complex, i.e., *Pinus mugo*, *P. rotundata* and *P. uncinata*. The obtained results revealed a high conservation of their chloroplast genomes in terms of length, structure and number of genes. We confirmed very close relationships between these three taxa using inference and phylogenetic trees topology in which *P. mugo*, *P. rotundata* and *P. uncinata* form one distinct clade within the genus *Pinus* with strong support. Highly variable regions and distinct microsatellite loci patterns have been identified in the genomes of chloroplast members of the *Pinus mugo* complex that could potentially be used in the future to discriminate and identify these taxa. Our analyses increase the knowledge of the *Pinus mugo* complex phylogeny and provide a valuable genomic baseline for future research into the evolutionary history and conservation of this highly polymorphic and enigmatic group, as well as the Pinaceae family in general. 

## Figures and Tables

**Table 1:** Basic features of chloroplast genomes among the seven taxa of Pinaceae.

Genome Features	*Pinus mugo*	*Pinus rotundata*	*Pinus uncinata*	*Pinus sylvestris*	*Pinus densiflora*	*Larix decidua*	*Abies alba*
Genome size (bp)	119,765	119,759	119,780	119,758	119,875	122,747	121,243
Total coding length (bp)	67,592	67,593	67,592	67,625	67,684	68,621	67,983
Protein coding length (bp)	60,339	60,339	60,339	60,384	60,444	61,524	60,810
rRNA coding length (bp)	4517	4518	4518	4518	4518	4520	4522
tRNA coding length (bp)	2736	2736	2735	2654	2723	2577	2651
Total GC content (%)	38.5	38.5	38.5	38.5	38.5	38.8	38.3
Total number of genes	121	121	121	116	118	110	113
Number of protein-coding genes	73	73	73	73	73	72	74
Number of rRNA genes	4	4	4	4	4	4	4
Number of tRNA genes	36	36	36	35	36	34	35
GenBank Acc. No.	MZ333466	MZ333465	MZ333464	KR476379	MK285358	AB501189	NC_042410

**Table 2:** Estimates of evolutionary divergence between four *Pinus* species. The number of base differences per site from between sequences are shown. Standard error estimate(s) are shown above the diagonal and were obtained by a bootstrap procedure (1000 replicates). This analysis involved four nucleotide sequences. All ambiguous positions were removed for each sequence pair (pairwise deletion option). There were a total of 120279 positions in the final dataset. Evolutionary analyses were conducted in MEGA X [48].

	*P. mugo*	*P. rotundata*	*P. uncinata*	*P. sylvestris*
*P. mugo*	-	0.000044	0.000055	0.000158
*P. rotundata*	0.000259	-	0.000061	0.000158
*P. uncinata*	0.000351	0.000409	-	0.000158
*P. sylvestris*	0.003117	0.003126	0.003184	-

**Table 3:** Estimates Simple sequence repeats (SSRs) identified in the *P. mugo*, *P. rotundata*, *P. uncinata* and *P. sylvestris* chloroplast genomes.

Taxon	ID	Type	Repeat Motif	Length (bp)	Start	End	Location	ID	Type	Repeat Motif	Length (bp)	Start	End	Location
*P. mugo*	1	p1	(C)12	12	15142	15153	IGS	11	p1	(A)11	11	79749	79759	IGS
	2	p1	(A)12	12	26050	26061	IGS	12	p1	(T)10	10	87077	87086	IGS
	3	c	(A)10(G)10	20	30198	30217	IGS	13	p1	(A)10	10	100605	100614	IGS
	4	p1	(T)23	23	40994	41016	IGS	14	p1	(T)10	10	103575	103584	IGS
	5	p1	(T)13	13	44949	44961	IGS	15	p1	(G)11	11	104142	104152	CDS (*ndhD*)
	6	p1	(T)10	10	48132	48141	IGS	16	p1	(A)13	13	106928	106940	IGS
	7	p1	(A)10	10	54429	54438	IGS	17	p1	(T)11	11	107335	107345	CDS (*rpl32*)
	8	p1	(A)10	10	67826	67835	IGS	18	p1	(A)10	10	109379	109388	IGS
	9	p1	(T)11	11	71751	71761	CDS (*ycf3*)	19	p1	(A)10	10	109840	109849	CDS (*rps12*)
	10	p2	(AT)6	12	73254	73265	IGS	20	p2	(AT)6	12	111752	111763	IGS
	1	p1	(C)13	13	15141	15153	IGS	11	p1	(T)11	11	87069	87079	IGS
	2	p1	(A)12	12	26050	26061	IGS	12	p1	(A)10	10	100597	100606	IGS
*P. rotundata*	3	c	(A)11(G)10	21	30197	30217	IGS	13	p1	(T)10	10	100883	100892	IGS
	4	p1	(T)15	15	40993	41007	IGS	14	p1	(T)10	10	103568	103577	IGS
	5	p1	(T)15	15	44940	44954	IGS	15	p1	(G)10	10	104135	104144	CDS (*ndhD*)
	6	p1	(T)11	11	48122	48132	IGS	16	p1	(A)13	13	106920	106932	IGS
	7	p1	(A)10	10	67816	67825	IGS	17	p1	(T)11	11	107327	107337	CDS (rpl32)
	8	p1	(T)12	12	71741	71752	CDS (*ycf3*)	18	p1	(A)10	10	109375	109384	IGS
	9	p2	(AT)6	12	73245	73256	IGS	19	p1	(A)10	10	109836	109845	CDS (*rps12*)
	10	p1	(A)11	11	79740	79750	IGS	20	p2	(AT)6	12	111746	111757	IGS
	1	p1	(C)13	13	15142	15154	IGS	11	p1	(T)11	11	87091	87101	IGS
	2	p1	(A)15	15	26051	26065	IGS	12	p1	(A)10	10	100620	100629	IGS
	3	c	(A)11(G)10	21	30203	30223	IGS	13	p1	(T)10	10	103590	103599	IGS
	4	p1	(T)23	23	40998	41020	IGS	14	p1	(G)10	10	104157	104166	CDS (*ndhD*)
*P. uncinata*	5	p1	(T)11	11	48131	48141	IGS	15	p1	(A)13	13	106942	106954	IGS
	6	p1	(A)10	10	54428	54437	IGS	16	p1	(T)11	11	107349	107359	CDS (*rpl32*)
	7	p1	(A)10	10	67836	67845	IGS	17	p1	(A)11	11	109393	109403	IGS
	8	p1	(T)13	13	71760	71772	CDS (*ycf3*)	18	p1	(A)11	11	109855	109865	CDS (*rps12*)
	9	p2	(AT)6	12	73265	73276	IGS	19	p2	(AT)6	12	111767	111778	IGS
	10	p1	(A)12	12	79761	79772	IGS							
	1	p1	(T)11	11	1376	1386	IGS	12	p1	(A)10	10	79947	79956	IGS
	2	p1	(A)10	10	9837	9846	IGS	13	p1	(T)10	10	87277	87286	IGS
	3	c	(C)10(T)11	21	15195	15215	IGS	14	p1	(A)10	10	100844	100853	IGS
	4	p1	(A)12	12	26112	26123	IGS	15	p1	(T)11	11	101130	101140	IGS
*P. sylvestris*	5	c	(A)11(G)10	21	30269	30289	IGS	16	p1	(T)10	10	101833	101842	CDS (*ndhH*)
	6	p1	(T)11	11	41059	41069	IGS	17	p1	(T)10	10	102658	102667	IGS
	7	p1	(T)19	19	45043	45061	IGS	18	p1	(G)11	11	104388	104398	CDS (*ndhD*)
	8	p1	(A)12	12	68030	68041	IGS	19	p1	(T)11	11	107567	107577	CDS (*rpl32*)
	9	p1	(T)14	14	71957	71970	CDS (*ycf3*)	20	p1	(A)10	10	109610	109619	IGS
	10	p2	(AT)6	12	73462	73473	IGS	21	p1	(A)12	12	110071	110082	CDS (*rps12*)
	11	p2	(AT)6	12	79134	79145	IGS	22	p2	(AT)7	14	111984	111997	IGS

c, compound SSR; p1, mono-nucleotide SSR; p2, di-nucleotide SSR.

**Table 4:** GenBank information on complete chloroplast genomes of conifer taxa used in phylogenetic analyses in this study.

GenBank Accession	Taxon	Common Name	Family
NC_042410	*Abies alba*	silver fir	Pinaceae
KP742350	*Abies koreana*	Korean fir	Pinaceae
AB501189	*Larix decidua*	common larch	Pinaceae
NC_036811	*Larix sibirica*	Siberian larch	Pinaceae
NC_021456	*Picea abies*	Norway spruce	Pinaceae
NC_032367	*Picea asperata*	dragon spruce	Pinaceae
MN536531	*Pinus cembra*	Swiss stone pine	Pinaceae
MK285358	*Pinus densiflora*	Japanese red pine	Pinaceae
MZ333466	*Pinus mugo* subsp. *mugo*	dwarf mountain pine	Pinaceae
MZ333465	*Pinus mugo* subsp. *rotundata*	peat-bog pine	Pinaceae
MZ333464	*Pinus mugo* subsp. *uncinata*	mountain pine	Pinaceae
NC_039585	*Pinus pinea*	Italian stone pine	Pinaceae
NC_026302	*Pinus strobus*	Eastern white pine	Pinaceae
KR476379	*Pinus sylvestris*	Scots pine	Pinaceae
KY964286	*Pinus taeda*	loblolly pine	Pinaceae
MH536745	*Podocarpus latifolius*	broad-leaved yellowwood	Podocarpaceae

## Data Availability

Data is contained within the article.

## Supplementary Materials

The following are available online at https://www.mdpi.com/article/10.3390/plants10071331/s1, Table S1: List of genes annotated in the chloroplast genomes of P. mugo, P. rotundata and P. uncinata sequenced in this study.
