# Supplementary material for: New Insight into Taxonomy of European Mountain Pines, Pinus mugo Complex, Based on Complete Chloroplast Genomes Sequencing
Source: Plants (Basel). 2021 Jun 29;10(7):1331. doi: 10.3390/plants10071331 (PMC8309040; doi:10.3390/plants10071331)
Supplement: Supplementary file 1 [file plants-10-01331-s001.zip › plants-1267297-supplementary.pdf]

Supplementary Table S1

Supplementary Table S1. List of genes annotated in the chloroplast genomes of *P. mugo*, *P. rotundata* and *P. uncinata* sequenced in this study.

No.	Classification of genes	Name of genes	Number
1	Photosystem I	*psaA, psaB, psaC, psaI, psaJ, psaM(x2), ycf3, ycf4*	9
2	Photosystem II	*psbA, psbB, psbC, psbD, psbE, psbF, psbH, psbI, psbJ, psbK, psbL, psbM, psbN, psbT, psbZ*	15
3	Cytochrome b/f complex	*petA, petB, petD, petG, petL, petN*	6
4	ATP synthase	*atpA, atpB, atpE, atpF, atpH, atpI*	6
5	NADH dehydrogenase	*ndhB, ndhC, ndhD, ndhE, ndhH, ndhI, ndhK*	7
6	RubisCO large subunit	*rbcL*	1
7	RNA polymerase	*rpoA, rpoB, rpoC1, rpoC2*	4
8	Ribosomal proteins – small units (SSU)	*rps2, rps3, rps4, rps7, rps8, rps11, rps12, rps14, rps15, rps18, rps19*	11
9	Ribosomal proteins – large units (LSU)	*rpl2, rpl14, rpl16, rpl20, rpl22, rpl23, rpl32, rpl33, rpl36*	9
10	Other genes/Miscellaneous	*accD, ccsA, cemA, chlB, chlL, chlN, clpP, infA, matK*	9
11	Protein of unknown function / Hypothetical chloroplast reading frames	*ycf1, ycf2, ycf12, ycf68*	4
12	Transfer RNAs	*trnA-UGC, trnC-GCA, trnD-GUC, trnE-UUC, trnF-GAA, trnfM-CAU, trnG-GCC, trnG-UCC, trnH-GUG* (x2), *trnI-GAU, trnK-UUU, trnL-CAA, trnL-UAG, trnL-UAA, trnM-CAU* (x3), *trnN-GUU, trnP-GGG, trnP-UGG, trnQ-UUG, trnR-ACG, trnR-CCG, trnR-UCU, trnS-UGA, trnS-GGA, trnS-GCU* (x2), *trnT-UGU, trnT-GGU, trnV-GAC* (x2), *trnV-UAC, trnW-CCA, trnY-GUA*	36
13	Ribosomal RNAs	*rrn4*.5, *rrn5*, *rrn16*, *rrn23*	4
	**Total**		**121**

(x2) indicates that the number of the repeat unit is 2
